# Ethical Challenges in Managing a Displaced Neer III Humeral Head Fracture in an Undocumented Immigrant With Early Alzheimer’s Disease: A Case Report

**DOI:** 10.7759/cureus.96805

**Published:** 2025-11-13

**Authors:** Albert Gassull

**Affiliations:** 1 Emergency Medicine, Hospital Sant Joan de Reus, Tarragona, ESP

**Keywords:** alzheimer’s disease, delphi consensus, early-onset dementia, falls, fracture fixation, health care access, osteoporotic fracture, proximal humerus fracture, undocumented migrants, universal health coverage

## Abstract

This report describes an 82-year-old undocumented Peruvian female immigrant presenting to the ED with right elbow pain and limited shoulder mobility, diagnosed with a displaced Neer III right humeral head fracture sustained two days prior. She had not clearly communicated shoulder pain to her family for two days post-injury, possibly due to early Alzheimer’s disease, which may have impaired her ability to recognize or articulate the severity of her injury, illustrating the clinical-ethical intersection. Radiographic imaging confirmed the fracture with medial displacement, necessitating surgical intervention. Due to her undocumented status and lack of health insurance, she was required to pay the full cost of a reverse shoulder prosthesis (€24,000), which she could not afford. Her Alzheimer’s-related dependency and absence of family support in Peru prevented seeking treatment abroad, resulting in discharge with a sling. Comprehensive blood tests ruled out metabolic contributors to her fall. This case highlights ethical challenges in healthcare access for undocumented immigrants, compounded by Alzheimer’s-related communication and dependency issues, underscoring disparities and the need for equitable care. This case exemplifies how clinical vulnerability and legal status can intersect to create barriers in emergency and trauma care.

## Introduction

Proximal humerus fractures, including Neer III type, are prevalent in elderly populations, typically arising from low-energy falls, and displaced cases often require surgical management such as reverse shoulder arthroplasty to regain function [1]. Undocumented immigrants encounter substantial systemic barriers, including lack of insurance coverage, administrative restrictions, financial limitations, and fear of deportation, which impede access to essential treatments and pose ethical questions regarding healthcare equity, particularly in minority health contexts [2]. Furthermore, early Alzheimer’s disease adds layers of complexity by hindering communication, autonomy, and timely medical presentation, potentially delaying diagnosis and complicating treatment decisions [3]. In emergency settings, routine diagnostics like comprehensive blood panels and radiographic imaging serve to rule out underlying systemic causes of falls, offering an equitable and cost-effective approach to evaluation that aligns with principles of accessible care for vulnerable populations. This case report details a displaced Neer III humeral head fracture in an undocumented immigrant with early Alzheimer’s disease, highlighting the intertwined clinical and ethical challenges in ensuring equitable care for such vulnerable groups, as explored in the discussion on systemic inequities and the need for reform.

## Case presentation

An 82-year-old Peruvian female, an undocumented immigrant, presented to the ED with right elbow pain persisting for two days following a fall at home. She reported limited right shoulder mobility but had not clearly communicated shoulder pain to her family for two days post-injury, possibly due to early Alzheimer’s disease, diagnosed six months prior. The Mini-Mental State Examination score was 24/30 (administered by the neuropsychology unit of Institut Pere Mata during her diagnostic evaluation; only the score is reported in this case report) [4]. She lived with her daughter in Spain and had no family in Peru, making travel for treatment unfeasible given her cognitive impairment and dependency on her daughter for daily support. No neurological deficits were noted beyond her known cognitive impairment. Her medical history included early Alzheimer’s disease, with no other relevant past interventions or outcomes reported. Family history was unremarkable, and psychosocial history highlighted her undocumented status and reliance on family in Spain.

Timeline

The patient was diagnosed with early Alzheimer’s disease six months prior to presentation. She sustained a fall at home two days before presenting to the ED, with right elbow pain and limited shoulder mobility noted from the time of injury. Radiographic imaging and comprehensive blood testing were performed on the day of presentation. Orthopedic consultation occurred shortly thereafter, followed by discharge with a sling on the same day.

Diagnostic assessment

**Figure 1:**
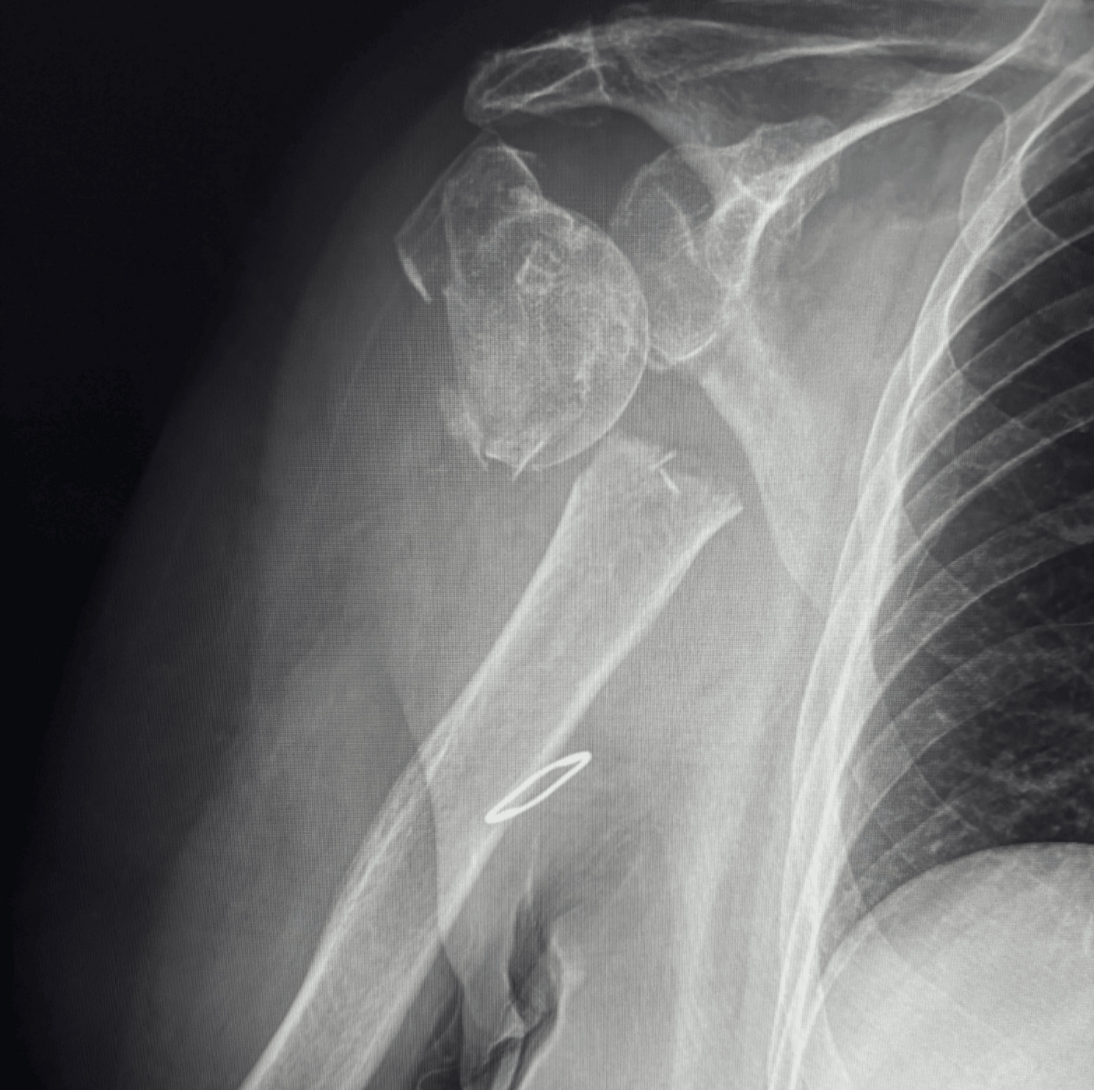
Radiograph of a proximal humerus fracture

Diagnostic methods included physical examination, radiographic imaging, and laboratory testing (comprehensive blood panel). Radiographs (Figure 1) showed a normal right elbow but a displaced right humeral head fracture with medial displacement, classified as a Neer III fracture, likely sustained two days prior based on her history [5].

A comprehensive blood panel was performed to rule out acute metabolic contributors to her recent fall (Table 1). While a single time-point test cannot definitively exclude chronic dysfunctions from months prior, the normal results effectively eliminated acute imbalances such as hypoglycemia, electrolyte disturbances, or hypothyroidism that could have precipitated the fall.

**Table 1:** Comprehensive blood analysis ALP, alkaline phosphatase; ALT, alanine aminotransferase; AST, aspartate aminotransferase; BUN, blood urea nitrogen; TSH, thyroid-stimulating hormone

Section	Test	Result	Reference range	Purpose
Hematology	Hemoglobin	12.8 g/dL	12.0-15.5 g/dL	Rule out anemia as a cause of weakness or falls
	Hematocrit	38%	36-46%	Assess for anemia or dehydration
	White blood cells	6.5 × 10³/µL	4.0-11.0 × 10³/µL	Rule out infection or inflammation
	Platelets	220 × 10³/µL	150-450 × 10³/µL	Assess clotting ability
Electrolytes	Sodium	138 mmol/L	135-145 mmol/L	Rule out hyponatremia or hypernatremia causing confusion or weakness
	Potassium	4.2 mmol/L	3.5-5.0 mmol/L	Rule out hypo- or hyperkalemia causing muscle dysfunction
	Calcium	9.1 mg/dL	8.5-10.2 mg/dL	Rule out hypocalcemia contributing to falls
	Magnesium	2.0 mg/dL	1.7-2.2 mg/dL	Rule out hypomagnesemia causing muscle weakness
Metabolic	Glucose	85 mg/dL	70-110 mg/dL	Rule out hypoglycemia as a cause of the fall
	Creatinine	0.8 mg/dL	0.6-1.1 mg/dL	Assess kidney function
	Blood urea nitrogen	15 mg/dL	7-20 mg/dL	Assess kidney function and hydration
Liver function	ALT	25 U/L	7-56 U/L	Rule out liver dysfunction contributing to weakness
	AST	20 U/L	10-40 U/L	Rule out liver or muscle damage
	ALP	70 U/L	44-147 U/L	Assess for bone or liver pathology
Thyroid function	TSH	2.3 mIU/L	0.4-4.0 mIU/L	Rule out hypothyroidism-related weakness
Inflammatory markers	C-reactive protein	0.5 mg/L	<5 mg/L	Rule out acute inflammation or infection

Diagnostic challenges included the patient’s delayed communication of symptoms, possibly due to cognitive impairment. The primary diagnosis was a Neer III humeral head fracture; no other diagnoses were considered. Prognostic characteristics include the potential for poor functional recovery without surgery.

Therapeutic intervention

Orthopedic consultation recommended a reverse shoulder prosthesis (surgical intervention), the cost of which (€24,000) exceeded the patient’s means due to her undocumented status and lack of insurance coverage [6]. Returning to Peru was not viable due to her Alzheimer’s-related dependency and absence of family support. No surgical intervention occurred; instead, conservative management with a sling was implemented. No pharmacologic interventions were detailed. Changes in therapeutic interventions included shifting from proposed surgery to conservative care due to financial constraints.

Follow-up and outcomes

The patient was discharged with a sling and referred to social services for financial aid exploration. Follow-up was arranged with a primary care provider. Clinician-assessed outcomes included conservative management without surgery; patient-assessed outcomes were not directly available due to cognitive impairment. No important follow-up test results were reported. Intervention adherence and tolerability were not assessed in detail, as the patient was discharged with basic instructions. No adverse or unanticipated events were noted.

## Discussion

This case highlights the medical and ethical complexities of managing a displaced Neer III humeral head fracture in an undocumented immigrant with early Alzheimer’s disease. The fracture, sustained two days prior but not clearly communicated due to Alzheimer’s-related cognitive impairment, required surgical intervention to optimize shoulder function [1]. However, the patient’s undocumented status and financial constraints led to conservative management with a sling, which is associated with poor outcomes, including persistent pain and limited mobility [7]. Her Alzheimer’s disease, causing dependency on her daughter and precluding travel to Peru due to lack of family support, heightened her vulnerability [4].

The comprehensive blood panel (Table 1) ruled out acute metabolic contributors to her fall, such as hypoglycemia, electrolyte imbalances, or hypothyroidism, which are critical to assess in older adults with trauma [8]. While a single time-point test cannot definitively exclude chronic dysfunctions from months prior, the normal results suggested the fall was mechanical, likely related to the fracture, underscoring the impact of delayed reporting, possibly due to Alzheimer’s-related communication difficulties [3]. These tests, feasible in emergency settings, ensured a thorough evaluation without specialized diagnostics.

Ethically, this case challenges justice and beneficence. Justice demands equitable access to care, yet the €24,000 cost of the prosthesis created an insurmountable barrier, effectively denying surgery [2]. Beneficence may have been limited by discharging her with a sling, which fails to address the long-term morbidity of an untreated displaced fracture [6]. Her Alzheimer’s disease amplifies these concerns, as her impaired communication and dependency necessitate robust advocacy, inadequately provided by the offer to return to Peru or pay an unaffordable sum [4]. This case reflects systemic inequities faced by undocumented immigrants, particularly those with cognitive impairments, including fear of legal repercussions, limited access to public funds, and cultural barriers that exacerbate treatment difficulties [9]. This aligns with existing literature on similar ethical cases where financial constraints lead to suboptimal outcomes in vulnerable populations.

Strengths of the approach include the use of accessible diagnostics to rule out contributory factors. Limitations involve the inability to provide surgical intervention due to systemic barriers. The rationale for conclusions emphasizes the intersection of clinical needs and ethical principles in emergency care. The primary takeaway lessons from this case report are the ethical imperatives for healthcare equity in undocumented populations with cognitive impairments and the value of routine diagnostics in resource-limited scenarios.

Healthcare systems must implement solutions like subsidized surgical programs or enhanced social service integration, promoting interdisciplinary collaboration between orthopedic, social work, and public health teams to mitigate disparities [2]. Clinicians should leverage accessible diagnostics, as demonstrated, to ensure comprehensive care within emergency constraints. This case underscores the need for systemic reform to ensure equitable treatment for vulnerable populations.

Patient perspective

Due to the patient’s early Alzheimer’s disease and associated cognitive impairment, she was unable to directly share her perspective on the treatments received.

Informed consent

Informed consent for publication of this case report was obtained from the patient’s daughter, who serves as her legal guardian.

## Conclusions

This case illustrates the challenges of managing a displaced Neer III humeral head fracture in an undocumented immigrant with early Alzheimer’s disease. Delayed pain communication, possibly due to Alzheimer’s, and financial barriers prevented surgical intervention, raising ethical concerns about healthcare equity. Routine blood tests ruled out systemic causes of her fall. Systemic reforms and cross-sector coordination are essential to ensure equitable acute and post-acute care for vulnerable populations. Emergency physicians can play a key role in advocating for these patients through policy change and resource allocation.

## References

[1] Williams AA, Dey Hazra RO, Dornan G (2025). Consensus statement on the treatment of proximal humerus fractures: a Delphi approach by the Neer Circle of the American Shoulder and Elbow Surgeons. J Shoulder Elbow Surg.

[2] Aljadeeah S, Payedimarri AB, Kielmann K, Michielsen J, Wirtz VJ, Ravinetto R (2024). Access to medicines among asylum seekers, refugees and undocumented migrants across the migratory cycle in Europe: a scoping review. BMJ Glob Health.

[3] Zhou BN, Zhang Q, Li M (2023). Alzheimer's disease and its associated risk of bone fractures: a narrative review. Front Endocrinol (Lausanne).

[4] Dev K, Javed A, Bai P, Murlidhar Murlidhar, Memon S, Alam O, Batool Z (2021). Prevalence of falls and fractures in Alzheimer's patients compared to general population. Cureus.

[5] Kristiansen B, Andersen UL, Olsen CA, Varmarken JE (1988). The Neer classification of fractures of the proximal humerus. An assessment of interobserver variation. Skeletal Radiol.

[6] Cheng B, Jiang X, Zhang X (2024). Biomechanical study of two different fixation methods for the treatment of Neer III proximal humerus fractures. BMC Musculoskelet Disord.

[7] Matsumoto S, Hosoi T, Yakabe M (2024). Early-onset dementia and risk of hip fracture and major osteoporotic fractures. Alzheimers Dement.

[8] Hacker K, Anies M, Folb BL, Zallman L (2015). Barriers to health care for undocumented immigrants: a literature review. Risk Manag Healthc Policy.

[9] Stevenson K, Antia K, Burns R (2024). Universal health coverage for undocumented migrants in the WHO European region: a long way to go. Lancet Reg Health Eur.

